# Iatrogenic post-surgical tension pneumosyrinx—a first reported case

**DOI:** 10.1007/s00701-022-05286-8

**Published:** 2022-06-27

**Authors:** Shyam S. Swarna, Josephine Jung, Steve Connor, Maurizio Belci, Gordon Grahovac

**Affiliations:** 1 National Spinal Injuries Centre, Stoke Mandeville Hospital, Aylesbury, UK; 2 Department of Neurosurgery, King’s College Hospital NHS Foundation Trust, Denmark Hill, London, SE5 9RS UK; 3 Institute of Psychiatry, Psychology and Neurosciences, King’s College London, London, UK; 4 Department of Neuroradiology, King’s College Hospital, London, UK

**Keywords:** Iatrogenic, Pneumosyrinx, Pneumorrhachis, Spinal cord tumour, Tension

## Abstract

We describe the first ever-reported occurrence of a post-operative tension pneumosyrinx occurring after a resection of an intradural intramedullary spinal tumour in a 40-year-old patient. Post-operatively, he developed sudden onset paraplegia and imaging revealed a tension pneumosyrinx which was subsequently surgically decompressed. He made a gradual neurological recovery. This is an extremely rare complication with potentially long-lasting deleterious effects on patients’ neurological status if not recognized. We aim to bring this pathology to the attention of our neurosurgical colleagues and share our surgical approach and management to assist those who may encounter this pathology in the future.

## Introduction

Pneumosyrinx is a rare phenomenon defined as migration of air within the central canal of the spinal cord. In this case report, we discuss the presentation of a patient with pneumosyrinx and review the existing literature on pneumorrhachis which is a condition closely associated with this. The authors describe the case of a 40-year-old who presented with an acute neurological deterioration after resection of a cervico-thoracic intradural intramedullary (IDIM) tumour due to severe tension pneumosyrinx caused by CSF entrapment in the arachnoid space. To the authors’ knowledge, this is the first case of iatrogenic/post-surgical tension pneumosyrinx to be described in the literature. The patient required a return to theatre for exploration of the cervico-thoracic wound and release of the adhesive arachnoid tissue which was causing a one-way valve effect and compression of the spinal cord.

## Case presentation

**Fig. 1:**
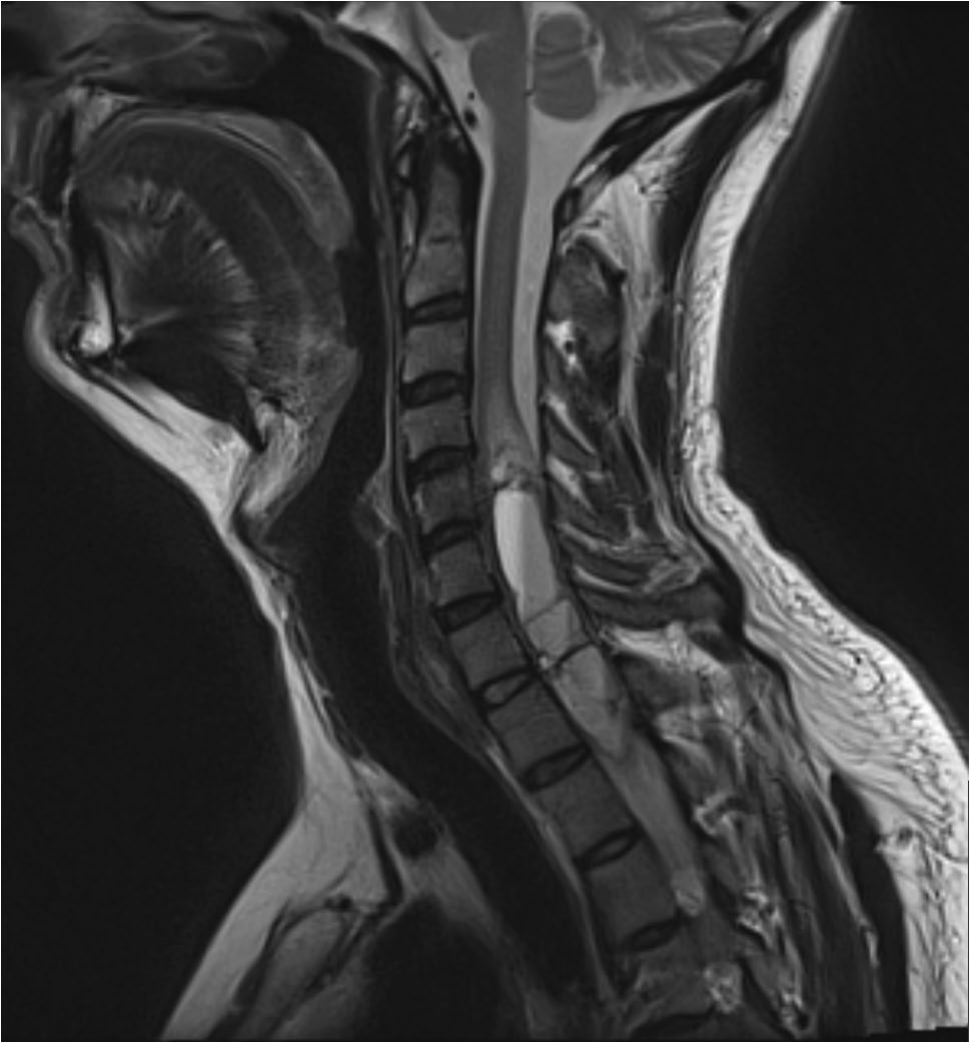
Pre-operative T2-weighted sagittal MRI of the cervical spine showing a hyperintense cystic intradural intramedullary lesion spanning from C4 to T2 and expanding the cord

A 40-year-old male presented with a 3-year history of paraesthesia mainly affecting the fingers in his right hand. Magnetic resonance imaging (MRI) of the cervical spine revealed a cervico-thoracic IDIM tumour spanning from the C4 to T2 level (Fig. 1).

The patient underwent a laminectomy for complete tumour resection with intra-operative neuromonitoring (IONM). The myelotomy was performed on the dorsal midline of the spinal cord, immediately identifying the superficial tumour which was encased by the thin spinal cord. The tumour (from C3 down to T2) was carefully dissected with the cystic component drained (no excessive amounts of CSF were drained during this procedure). The myelon was not sutured during the initial surgery and dural closure was performed with 5.0 prolene with copious irrigation. Post-operatively, the patient displayed a new-onset mild right grip and right leg weakness with 4 out of 5 power on the Medical Research Council (MRC) scale in line with IONM findings. Ten hours after surgery, the patient started complaining of increased neck pain followed by haemodynamical instability and development of complete loss of power in both lower limbs and the right upper limb (0 out of 5 on MRC) with only preserved shoulder shrug bilaterally (4 out of 5 on MRC) and left biceps flexion (3 out of 5 on MRC). He was also insensate to the indwelling urinary catheter.

**Fig. 2:**
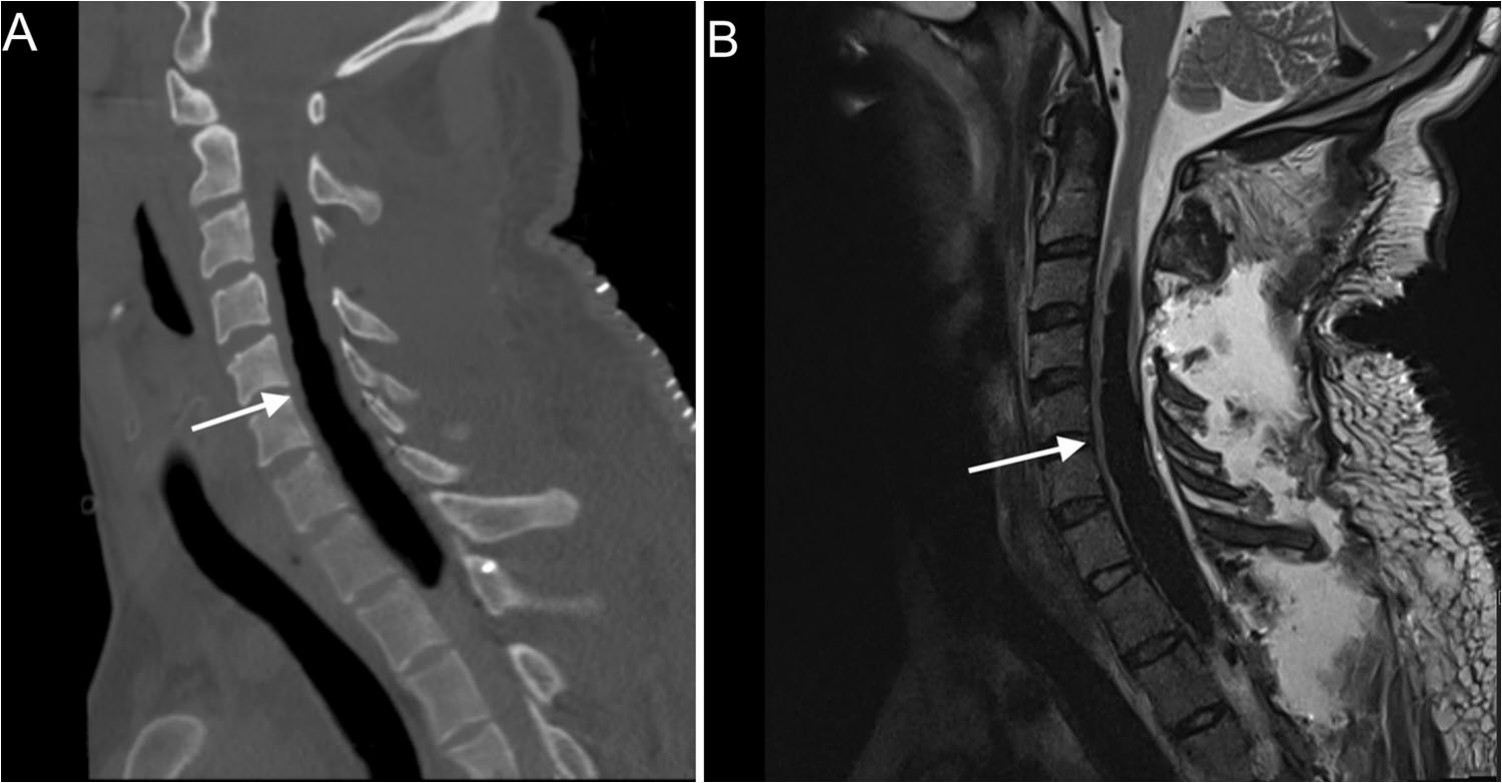
**A** Post-operative cervical sagittal CT demonstrating a tension pneumosyrinx extending from C3 to the level of T2; **B** Post-operative sagittal T2-weighted MRI of the cervical spine demonstrating a hypointense lesion at the same level which is consistent with a tension pneumosyrinx

Post-operative computer tomography (CT) and MRI scan of the neck showed a long segment of tension pneumosyrinx (Fig. 2A and B) for which he subsequently underwent emergency re-exploration. CT brain imaging was normal and there was no pneumocephalus.

After opening of the dura, the cord herniated through the dural opening due to expansion of the arachnoid in front of the cord. After releasing the entrapped cerebrospinal fluid (CSF) from the ballooning arachnoid space in front of the cord (similar to an arachnoid cyst), it was decompressed and CSF flow re-established.

**Fig. 3:**
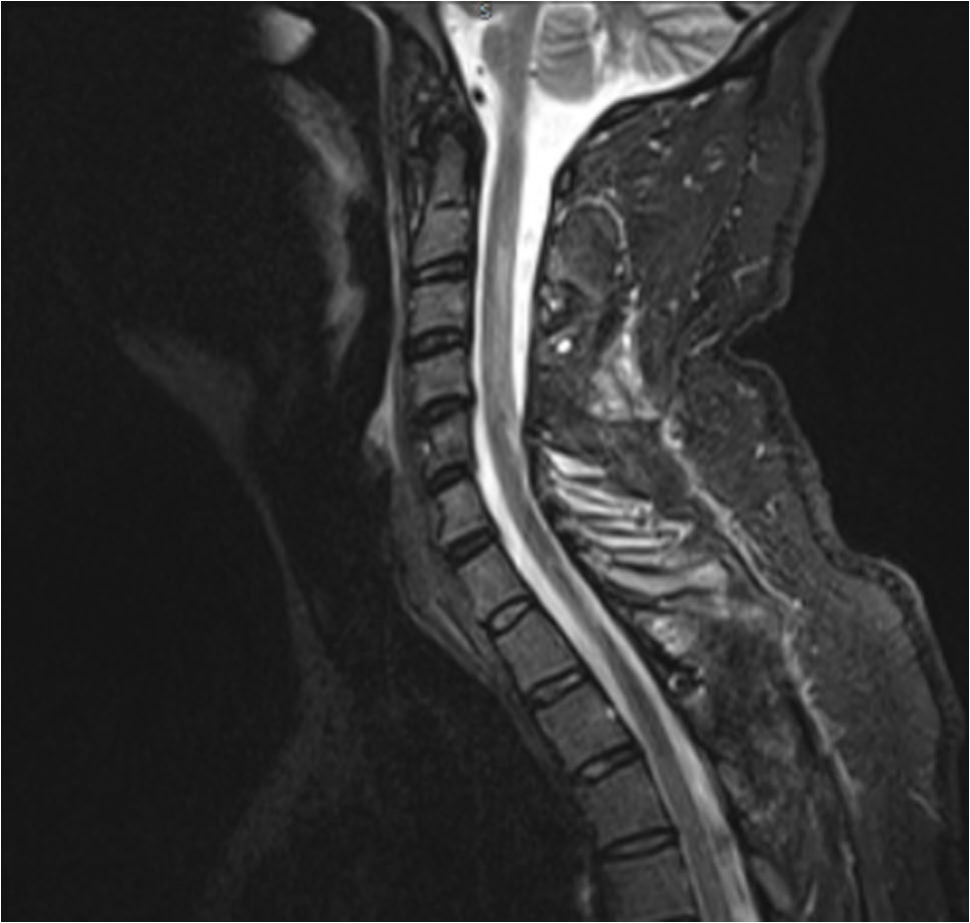
T2-weighted MRI scan 14 weeks post re-exploration following tension pneumosyrinx

The patient made a gradual recovery and was transferred for further neuro-rehabilitation. He had residual weakness involving the right arm and right lower limb with ongoing bladder and bowel dysfunction. Follow up MRI scan showed resolution of the tension pneumosyrinx (Fig. 3). The tumour was diagnosed as an ependymoma WHO grade II for which the patient did not require any further adjuvant treatment.

On admission to the tertiary neuro-rehabilitation unit, he had residual weakness predominantly affecting the right upper and lower limb with motor sparing on the left as well as ongoing sphincter dysfunction. His neurological level according to the International Standards for Neurological Classification of Spinal Cord Injury (ISNCSCI) was C2 ASIA Impairment Score D. During neuro-rehabilitation, he made excellent gains in all aspects of the Spinal Cord Independent Measure (SCIM) which includes self-care (feeding, grooming, bathing, and dressing), respiration and sphincter management, and patient mobility (transfers to/from bed and mobility indoors/outdoors). The SCIM on admission was 41 and subsequently improved to 86 at discharge. The patient also improved to independency with bladder and bowel function and was mobilizing with walking aids at 12 months post surgery.

## Discussion

Pneumosyrinx is an extremely rare complication. Upon search Pubmed, using the Boolean search term “pneumosyrinx,” there was only one article by Yanagawa et al. [19] which reported this condition. Pneumosyrinx was associated with pneumorrhachis. Pneumosyrinx is defined as entrapment of air or gas within the central canal of the spinal cord. Pneumorrhachis is the entrapment of air within the spinal canal. The previously published case report [5] describes a 26-year-old female who was involved in a road traffic accident. She was comatose on admission to the hospital and sadly died within 2 h of admission to hospital. Pneumosyrinx in this case was discovered on CT imaging of the thorax and abdomen and demonstrated air bubbles in the spinal canal between the second and fifth thoracic vertebrae. This was thought to be caused by migration of air into the central canal of the spinal cord. It was suspected that the existence of the pneumosyrinx could be an indirect sign of either spinal cord or medullary trauma with an open spinal canal injury, pneumocephalus, or pneumothorax. Pneumorrhachis was caused directly by the open injury. In other studies, direct open injury to the spinal canal, air migration into the spinal canal due to pneumomediastinum with barotrauma, or pneumothorax have been reported [3, 16, 19].

On Pubmed search using the Boolean search term “pneumorrhachis,” this was reported in 202 articles including both human and animal studies. Air in the spinal canal was first described by Gordon and Hardman in 1977 [9]. Pneumorrachis has been subclassified depending on the location of air which can be located either extradurally (epidural) or intradurally (in the subarachnoid space). The distinguishment of epidural pneumorrhachis is important as there are different pathophysiological mechanisms which may have different clinical implications and potential consequences. Pneumorrhachis can also occur in trauma without meningeal tears, with air simply accumulating in the epidural space [6]. The first report of epidural pneumorrhachis caused by blunt thoracic trauma was in a patient with bronchial rupture and pneumomediastinum following a road traffic accident [18]. The amount of air in epidural pneumorrhachis is generally small, as the driving pressure of the pneumothorax or pneumomediastinum is not high enough to push large volumes of air into the epidural space [6, 8]. The presence of pneumorrhachis signifies potential significant trauma, it however does not require specific treatment beyond the management of the precipitant such as a pneumothorax [5, 8, 10, 13].

In contrast, subarachnoid pneumorrhachis is usually associated with pneumocephalus [8]. The volume of air might raise intraspinal and by extension, intracranial pressure for which acute neurosurgical or interventional radiological treatment is required to facilitate drainage or to repair the dural tears [12, 20]. Less commonly, it may be atraumatic and cases have been reported where pneumorrhachis was due to violent coughing, forceful emesis, cardiopulmonary resuscitation, malignancy, infection, epidural anaesthesia [11], or marijuana or cocaine inhalation [1, 14]. Moreover, spontaneous pneumorrhachis has also been reported [7].

Some tumours are commonly associated with syringomyelia: two-thirds of haemangioblastomas and half of spinal ependymomas have an associated syrinx [2, 15, 17]. A possible explanation for syrinx development in association with IDIM tumours is that the cystic cavity is part of the tumour itself [17].

This is the second case of pneumosyrinx reported in the literature and the first one that is iatrogenic and associated with tension pneumosyrinx. In our patient, it was following the resection of an IDIM tumour. There are several possibilities regarding the mechanism for this to have happened. It could have occurred by the same mechanism leading to syringomyelia which is due to mechanisms affecting fluid dynamics referred to as “slosh” and “suck” [4]. The “slosh” effects are due to increased epidural venous flow triggered by activities such as coughing and sneezing. In this case, the potential culprit could have been positive pressure ventilation during the general anaesthetic as part of the operative procedure. The resection of the tumour formed a dead space within the cord structure thereby creating a vacuum effect. The second mechanism, “suck,” is the result of partial subarachnoid blockage. As the fluid is initially forced up due to increased epidural venous pressure, it returns slowly creating a pressure gradient across the partial subarachnoid block with negative pressure caudally to it [4]. Once the tumour was removed, however, venous congestion and therefore epidural pressure should have decreased and no excessive amount of positive pressure ventilation was used during the surgery. A second possibility is that air was entrapped during wound closure; however, the post-operative MRI cervicothoracic spine showing the tension pneumosyrinx did not reveal any air tracking along the surgical wound and no surgical drain was used. The third and most plausible hypothesis is that air was already entrapped intradurally during surgery. The head and cervical spine are often positioned lower than the thoracic spine during prone cervicothoracic surgery. During the tumour resection, air may have become entrapped in a distal part of the thoracic dural sack which was not visible at the time of dural closure. With a post-operative change in position from probe to supine and sitting up in bed, this air could have then migrated upwards and entered the large pial defect causing the gradual increase in pressure within the cord and resultant neurological deterioration. A mechanism involving entrapment of intradural air and downwards migration seems unlikely since the CT brain did not demonstrate any pneumocephalus.

## Conclusion

Pneumosyrinx is an extremely rare condition with potentially long-lasting deleterious effects on patients’ neurological status. We present the first case of post-operative tension pneumosyrinx to the spine. We aim to bring this pathology to the attention of our worldwide neurosurgical colleagues and share our surgical approach and management to assist those who may encounter this pathology in the future.

## Abbreviations

CSF: Cerebrospinal fluid
CT: Computer tomography
IDIM: Intradural intramedullary
IONM: Intra-operative neuromonitoring
ISNCSCI: International Standards for Neurological Classification of Spinal Cord Injury
MRI: Magnetic resonance imaging
MRC: Medical Research Council
SCIM: Spinal Cord Independent Measure
